# Referral patterns and attitudes of Primary Care Physicians towards chiropractors

**DOI:** 10.1186/1472-6882-6-5

**Published:** 2006-03-01

**Authors:** Barry R Greene, Monica Smith, Veerasathpurush Allareddy, Mitchell Haas

**Affiliations:** 1 Department of Health Management and Policy, College of Public Health, The University of Iowa, Iowa City, Iowa, USA; 2 Palmer Center for Chiropractic Research, Davenport, Iowa, USA; 3 Western States Chiropractic College, Portland, Oregon, USA

## Abstract

**Background:**

Despite the increasing usage and popularity of chiropractic care, there has been limited research conducted to examine the professional relationships between conventional trained primary care physicians (PCPs) and chiropractors (DCs). The objectives of our study were to contrast the intra-professional referral patterns among PCPs with referral patterns to DCs, and to identify predictors of PCP referral to DCs.

**Methods:**

We mailed a survey instrument to all practicing PCPs in the state of Iowa. Descriptive statistics were used to summarize their responses. Multivariable logistic regression analyses were conducted to identify demographic factors associated with inter-professional referral behaviors.

**Results:**

A total of 517 PCPs (33%) participated in the study. PCPs enjoyed strong intra-professional referral relationships with other PCPs. Although patients exhibited a great deal of interest in chiropractic care, PCPs were unlikely themselves to make formal referral relationships with DCs. PCPs in a private practice arrangement were more likely to exhibit positive referral attitudes towards DCs (p = 0.01).

**Conclusion:**

PCPs enjoy very good professional relationships with other PCPs. However, the lack of direct formalized referral relationships between PCPs and chiropractors has implications for efficiency, continuity, quality, and patient safety in the health care delivery system. Future research must focus on identifying facilitators and barriers for developing positive relationships between PCPs and chiropractors.

## Background

An increasing number of Americans are receiving health care from alternative care providers [1-3]. A study conducted by Eisenberg et al [1] demonstrated that more Americans are consulting alternate care providers than conventional allopathic trained physicians, and that 425 million visits were made to providers of unconventional therapy in 1990. This number far exceeded the estimated 388 million visits made to conventional primary-care physicians. A follow-up survey revealed that the number of visits to alternate practitioners increased from 425 million in 1990 to 629 million in 1997 [2]. Approximately 42% of Americans consulted at least 1 of 16 alternate care provider types. Chiropractic care was found to be one of the most frequently sought after alternative care, and evinced a high level of patient satisfaction and continuous utilization [4-6]. Despite evidence of increasing usage and popularity of chiropractic care in the United States, there is a dearth of research examining the professional relationships between chiropractors and conventional primary-care physicians (PCPs), namely medical doctors and osteopaths. To our knowledge very few studies have examined the professional relationships between physicians and alternate care providers [7,8]. The purpose of this study is to examine PCP referral patterns, intra-professional relationships among PCPs, inter-professional relationships between PCPs and chiropractors from the perspective of the PCP, and characteristics of PCPs that best predict referral behaviors towards chiropractors. Other study objectives were to examine the intra- and inter-professional relationships from the perspective of chiropractors; these reciprocal objectives, along with performance in focus groups on these topics will be published elsewhere.

## Methods

Drawing from information obtained during initial pilot groups of PCPs and chiropractors, we developed a survey instrument to measure patterns of referrals/consults and bidirectional communication between chiropractors and PCPs in Iowa. Specifically, we examined two dimensions of cross-disciplinary activities between the two practitioner types: sharing of patients (referrals), and sharing of patient information (clinical records). We examined both formal referrals and consults, as well as informal processes such as lay referrals and curbside consultations. We considered lay referrals as those in which patients were advised to contact the other practitioner on their own. Curbside consultation was explicitly defined within the survey instrument as "an informal process whereby a provider (typically an MD) obtains information or advice from another provider (typically an MD) to assist in the management of a particular patient, but the consultant neither reviews the patient's records nor examines the patient and does not document his/her recommendations" [9-13].

The survey instrument was pilot-tested on a convenience sample of PCP clinical faculty at University of Iowa – Carver College of Medicine, and their feedback was used to refine the instrument. The final survey instrument is appended as Appendix 1. The study was approved by The University of Iowa IRB.

We obtained the roster of MD and DO physicians from the database of the University of Iowa Office of Statewide Clinical Education Programs (OSCEP). OSCEP is an administrative unit within the College of Medicine. The OSCEP tracks all Iowa-licensed physicians, physician assistants, nurse practitioners, pharmacists, and dentists. The survey instrument was ground-mailed to all 1,561 MD's and DO's in the OSCEP list, with two follow-up mailed reminders to non-respondents.

We used chi-square tests and t-tests where appropriate to compare participants to non-participants. Simple descriptive statistics compare the responses of the 2 subgroups of PCPs: MD's and DO's. Chi-square tests were used to compare the referral patterns of PCPs to other PCPs and to Chiropractors.

Multivariable logistic regression models were used to identify variables that are most predictive of PCP relationships with chiropractors (DC). Separate logistic regression models were built to model each of six dichotomous outcome variables: 1) recommend patient see DC, 2) recommend patient contact DC, 3) make formal referral to DC, 4) receive referral from DC, 5) advise DC, and 6) receive advice from DC. The predictor variables used were sex (female is reference), MD degree (DO degree is reference), age, time since graduation, private practice (other practice set ups is reference, see table 1), internal medicine residency (family practice is reference), and metropolitan county (non-metropolitan is reference). The Hosmer and Lemeshow goodness-of-fit test was used to examine the adequacy of the multivariable models. A two-tailed p-value of less than 0.05 was deemed to be statistically significant for all the analyses. SAS version 9.1 (Cary, NC) and SPSS version 13.0 (Chicago, IL) were used for statistical analyses.

**Table 1:** Profile of Iowa PCPs

**Attribute**		**Participants (n = 513)**	**Non-Participants (n = 1099)**	**p-value**
Age	Mean (SD)	48.8 (10.2)	47.3 (9.5)	.004
Sex	Women	24.8%	26.6%	.465
	Men	75.2%	73.4%	
Degree	DO	21.2%	28.3%	.003
	MD	78.8%	71.7%	
Specialty	Family Practice	81.7%	74.2%	< 0.001
	Internal Medicine	18.3%	25.8%	
Activity	Private Practice	84.4%	85.4%	.525
	Others (Administration, teaching, research, public/community health, student health, and urgent care)	15.6%	14.6%	
Residency	Family Practice	64.3%	59.5%	.008
	Internal Medicine	17.7%	25.3%	
	Others	1.4%	1%	
	Unknown	16.6%	14.2%	
Years since graduation	Mean (SD)	21.2 (10.4)	19.9 (25)	.267
Practice Arrangement	HMO	0.4%	0.8%	.308
	Independent Practice	23.8%	21.5%	
	Hospital-based Independent Practice	1.0%	0.5%	
	Integrated Health System	55.9%	54.0%	
	Physician Network	8.8%	11.1%	
	Unknown	10.1%	12.1%	

PCP = Primary Care Physician

## Results

Five hundred seventeen physicians responded to the survey. This is 33% of the entire universe of 1,561 primary care physicians in the state of Iowa. These rates are comparable to that obtained in similar surveys of physicians and other health professionals [14,15]. After excluding ineligible surveys (i.e. respondents no longer in active practice), 513 PCPs were included in the analysis. The study group then consisted of 404 medical doctors and 109 osteopaths.

A profile comparison revealed only small differences between study participants and non-participants (Table 1). Respondents were less likely to be osteopaths, less likely to have specialized in internal medicine, and more likely to have completed a family practice residency.

### PCP intra-professional relationships

Table 2 summarizes the responses of medical doctors (MDs) and osteopaths (DOs) to questions that assessed their referral patterns to/from other PCPs. The results indicate that both DO's and MD's preferred to initiate formal referral to other PCPs (> 98%) rather than have the patient make the initial contact with the doctor. Over 70% of referring physicians sent a case report or clinical records "usually" or "always." Less than 10% of physicians "never" included a report with the referral. All physicians included an explanation for the referral at least "some of the time", and over 96% did so "routinely".

**Table 2:** Intra-professional relationships of PCPs

**Question**	**Response**	**DO N (%)**	**MD N (%)**
**Do you recommend patients contact doctor on own or initiate formal referral yourself?**	Patient contact doctor	2 (1.9%)	2 (0.5%)
	Doctor initiates referral	101 (98.1%)	370 (99.2%)
	Unknown	0	1 (0.3%)
**How often referral includes sending a case report?**	Always	20 (19.4%)	138 (36.5%)
	Usually	43 (41.7%)	142 (37.6%)
	Sometimes	30 (29.1%)	85 (22.5%)
	Never	10 (9.7%)	13 (3.4%)
**How often referral includes sending X-Rays or X-Ray report?**	Always	34 (33%)	105 (27.6%)
	Usually	44 (42.7%)	143 (37.6%)
	Sometimes	24 (23.3%)	129 (33.9%)
	Never	1 (1%)	3 (0.8%)
**How often referral includes sending clinical records other than X-Rays?**	Always	25 (24.3%)	100 (26.2%)
	Usually	47 (45.6%)	186 (48.8%)
	Sometimes	29 (28.2%)	90 (23.6%)
	Never	1 (1%)	3 (0.8%)
	Unknown	1 (1%)	2 (0.5%)
**How often referral includes sending reason for referrals?**	Always	81 (79.4%)	320 (84.4%)
	Usually	17 (16.7%)	52 (13.7%)
	Sometimes	4 (3.9%)	7 (1.8%)
**Have you accepted referral from other doctors?**	Yes	83 (79%)	313 (82.2%)
	No	22 (21%)	68 (17.8%)
**How often do you send clinical information to referring doctor as follow-up to referral?**	Always	44 (55%)	152 (48.7%)
	Usually	24 (30%)	102 (32.7%)
	Sometimes	9 (11.3%)	49 (15.7%)
	Never	3 (3.8%)	9 (2.9%)
**Has other PCP obtained clinical information or advice via curbside consultation**	Yes	103 (97.2%)	365 (94.1%)
	No	3 (2.8%)	23 (5.9%)
**Have you obtained clinical information or advice from another PCP via curbside consultation?**	Yes	102 (96.2)	375 (97.7%)
	No	4 (3.8%)	9 (2.3%)

PCP = Primary Care Physician

DO = Doctor of Osteopathy

MD = Medical Doctor

Seventy nine percent of DO's and 82% of MD's reported that they "always accepted" referrals from other PCPs (or peers), and only 4% of DO's and 3% of MD's "never" sent follow-up clinical information to referring physicians. PCPs did not accept referrals from physicians when they felt that caring for the referred patient was beyond their scope of expertise or were not covered by insurance.

Curbside consultation was common practice among both DO's and MD's. Physicians were about equally likely to receive or give a consultation. The referral patterns of both DO's and MD's were very similar.

### Inter-professional relationships

The results of the responses of PCPs to questions assessing their referral patterns to/from chiropractors are summarized in Table 3. Eighty-one percent of DO's and 87% of MD's reported that their patients had asked them for information about chiropractic, and close to 75% of PCPs have patients who have requested a referral to a chiropractor. Approximately 65% of DO's and MD's had recommended that their patients consult a chiropractor. However, only 24% of DO's and 29% of MD's had themselves formally referred a patient to a chiropractor. The common reasons for referring a patient to a chiropractor were back or neck pains, unresponsive chronic pain, fibromyalgias, and musculoskeletal conditions. A vast majority of both DO's and MD's preferred that their patients contact chiropractors on their own rather than the physicians initiating a formal referral themselves.

**Table 3:** Inter-professional relationships between PCPs and chiropractors

**Question**	**Response**	**DO N (%)**	**MD N (%)**
**Have you ever recommended patient see a DC?**	Yes	69 (65.7%)	251 (64.4%)
	No	36 (34.3%)	139 (35.6%)
**Do you recommend patient contacts DC on own or initiate formal referral?**	Patient contacts DC	61 (87.1%)	215 (87%)
	Doctor initiates referral	8 (11.4%)	29 (11.7%)
	Unknown	1 (1.4%)	3 (1.2%)
**Have you ever referred patient to DC for evaluation or treatment?**	Yes	22 (24.2%)	96 (29.4%)
	No	69 (75.8%)	230 (70.3%)
**How often referral includes sending case report?**	Always	2 (10%)	22 (23.9%)
	Usually	8 (40%)	21 (22.8%)
	Sometimes	7 (35%)	22 (23.9%)
	Never	3 (15%)	27 (29.3%)
**How often referral includes sending X-rays or X-ray report?**	Always	2 (9.5%)	15 (16.5%)
	Usually	8 (38.1%)	25 (27.5%)
	Sometimes	7 (33.3%)	31 (34.1%)
	Never	4 (19%)	20 (22%)
**How often referral includes sending clinical records other than X-rays?**	Always	1 (4.8%)	12 (13.6%)
	Usually	8 (38.1%)	19 (21.6%)
	Sometimes	7 (33.3%)	31 (35.2%)
	Never	5 (23.8%)	26 (29.5%)
**How often referral includes sending reason for referral?**	Always	12 (60%)	64 (73.6%)
	Usually	4 (20%)	9 (10.3%)
	Sometimes	2 (10%)	4 (4.6%)
	Never	2 (10%)	9 (10.3%)
**Have accepted referral from other DC**	Yes	85 (82.5%)	215 (55.4%)
	No	18 (17.5%)	173 (44.6%)
**How often do you send clinical information to referring DC as follow-up to referral?**	Always	18 (21.2%)	33 (15.9%)
	Usually	27 (31.8%)	49 (23.7%)
	Sometimes	25 (29.4%)	72 (34.8%)
	Never	15 (17.6%)	52 (25.1%)
**Has a DC obtained clinical information or advice via curbside consultation**	Yes	37 (34.9%)	71 (18.3%)
	No	69 (65.1%)	316 (81.7%)
**Have you obtained clinical information or advice from a DC via curbside consultation?**	Yes	12 (11.5%)	28 (7.3%)
	No	92 (88.5%)	356 (92.7%)

DC = Chiropractor

PCP = Primary Care Physician

While 82.5% of DO's accepted referral from a chiropractor only 55.4% of MD's accepted a referral from a chiropractor. The common reasons for not accepting a referral from chiropractors were the absence of a formal referral and health problems outside the PCP's area of expertise. Eighteen percent of DO's and 19% of MD's indicated that chiropractors did not send them any clinical information about the referred patient.

Thirty-five percent of DOs mentioned that a chiropractor obtained curbside advice from them. Only 18% of MD's mentioned that a chiropractor obtained curbside advice.

### Univariate analysis

The combined responses of DO's and MD's to questions assessing their referral behaviors to other PCPs and chiropractors are summarized in Table 4. Approximately 99% of the PCPs responded that they would initiate a patient referral to other PCPs, whereas only 12% said that they would initiate a referral to a chiropractor (p < 0.001). Close to 95% sent a case report when referring a patient to another PCP, but only 73% sent a case report when referring a patient to a chiropractor (p < 0.001). PCPs were also more likely to send X-rays or X-ray report and other clinical records to another PCP than to a chiropractor (p < 0.001).

**Table 4:** PCP referral patterns to other PCPs and DCs (Univariate analysis*)

**Question**	**Response**	**To/From PCPs N (%)**	**To/From DCs N (%)**	**p-value**
**Do you recommend patients contact doctor on own or initiate formal referral**	Patient contacts doctor	4 (0.8)	276 (88.2)	<.001
	Initiate referral	471 (99.2)	37 (11.8)	
**How often referral includes sending case report?**	Yes (at least sometimes)	458 (95.2)	82 (73.2)	<.001
	Never	23 (4.8)	30 (26.8)	
**How often referral includes sending X-Rays or X-Ray report?**	Yes (at least sometimes)	479 (99.2)	88 (78.6)	<.001
	Never	4 (0.8)	24 (21.4)	
**How often referral includes sending clinical records other than X-rays?**	Yes (at least sometimes)	477 (99.2)	78 (71.6)	<.001
	Never	4 (0.8)	31 (28.4)	
**How often referral includes sending reason for referral?**	Yes (at least sometimes)	481 (100)	95 (89.6)	<.001
	Never	0 (0)	11 (10.4)	
**Have you accepted referral from other doctors?**	Yes	396 (81.5)	300 (61.1)	<.001
	No	90 (18.5)	191 (38.9)	
**How often do you send clinical information to referring PCP/DC as follow-up to referral?**	Yes (at least sometimes)	380 (97)	224 (77)	<.001
	Never	12 (3)	67 (23)	
**Has other doctor obtained clinical information or advice via curbside consultation**	Yes	468 (94.7)	108 (21.9)	<.001
	No	26 (5.3)	385 (78.1)	
**Have you obtained clinical information or advice from another doctor via curbside consultation?**	Yes	477 (97.3)	40 (8.2)	<.001
	No	13 (2.7)	448 (91.8)	

* Chi-square tests.

DC = Chiropractor

PCP = Primary Care Physician

Eighty-one percent of PCPs had accepted a referral from another PCP but only 61% of PCPs had accepted a referral from a chiropractor (p < 0.001). PCPs were also not consistent in sending follow-up clinical information to referring chiropractors. Only 77% of PCPs who had accepted a referral from a chiropractor sent follow-up clinical information about the patient to the chiropractor, whereas 97% of PCPs who had accepted referral from another PCP sent follow-up clinical information to the referring physician (p < 0.001). Close to 95% of PCPs mentioned that other PCPs had obtained curbside clinical information or advice from them, whereas only 22% of PCPs mentioned that chiropractors had obtained curbside clinical advice from them (p < 0.001). PCPs were also more likely to obtain curbside clinical advice from other PCPs when compared to obtaining advice from chiropractors. Approximately 97% of PCPs mentioned that they had obtained curbside clinical advice from other PCPs, whereas only 8% of PCPs had obtained curbside clinical advice from chiropractors (p < 0.001).

### Multivariable analysis

Results from the Multivariable logistic regression analyses, including odds ratios and 95% confidence intervals for each predictor variable, are summarized in Table 5. All 6 models had a good fit. The first model shows that the odds that PCPs in private practice would recommend that their patients see a chiropractor were twice that of PCPs in other practice arrangements, after adjusting for other model predictors (p = 0.012). The next 2 models revealed no strong predictors of referral or type of referral to chiropractors. The 4^th ^model identified several predictors of accepting referrals from chiropractors: The odds of accepting referrals were 67% higher for men than women (p = 0.032), 3.7 times higher for DOs than MDs (p < 0.001), and 83% higher for PCPs in private practice than for those in other practice arrangements (p = 0.031). In Model 5, we found that the odds that an MD was approached by a chiropractor for curbside advice was only 47% of the odds that a DO would be approached (p = 0.004).

**Table 5:** PCP referral patterns to DCs (Multivariable analysis)

**Predictor Variables**	**Have you ever recommended patient see a DC? OR (95% CI)**	**Do you recommend patient contacts DC on own or initiate formal referral? OR (95% CI)**	**Have you ever referred patient to DC for evaluation or treatment? OR (95% CI)**	**Have you accepted referral from a DC? OR (95% CI)**	**Has a DC obtained clinical information or advice via curbside consultation OR (95% CI)**	**Have you obtained clinical information or advice from a DC via curbside consultation? OR (95% CI)**
Men	1.21 (0.75 – 1.93)	1.21 (0.53 – 2.79)	1.00 (0.58 – 1.73)	1.67 (1.04 – 2.67) ^¶^	1.80 (0.98 – 3.29)	0.63 (0.29 – 1.41)
MD	1.12 (0.69 – 1.82)	0.83 (0.34 – 2.02)	1.33 (0.75 – 2.35)	0.27 (0.15 – 0.49)^¶^	0.47 (0.28 – 0.79) ^¶^	0.67 (0.31 – 1.44)
Age	1.01 (0.94 – 1.07)	0.94 (0.85 – 1.03)	1.01 (0.94 – 1.08)	0.96 (0.90 – 1.02)	0.97 (0.91 – 1.04)	1.00 (0.91 – 1.10)
Time Since Graduation	0.97 (0.92 – 1.03)	1.07 (0.98 – 1.18)	0.99 (0.93 – 1.05)	1.03 (0.97 – 1.10)	1.01 (0.95 – 1.08)	0.99 (0.90 – 1.09)
Private Practice	2.00 (1.16 – 3.45)^¶^	1.65 (0.56 – 4.81)	1.58 (0.79 – 3.17)	1.83 (1.05 – 3.19) ^¶^	2.20 (0.92 – 5.24)	1.65 (0.45 – 6.01)
Internal Medicine	0.63 (0.39 – 1.02)	1.00 (0.37 – 2.67)	0.80 (0.43 – 1.47)	0.72 (0.44 – 1.18)	0.62 (0.31 – 1.21)	0.94 (0.37 – 2.38)
Metropolitan County	1.03 (0.68 – 1.57)	1.12 (0.51 – 2.44)	1.45 (0.90 – 2.31)	0.75 (0.49 – 1.14)	0.74 (0.45 – 1.21)	0.55 (0.25 – 1.17)
Number of cases in Final Model	495	313	417	491	493	488
Model-Fit (p-value)	0.12	0.48	0.09	0.96	0.78	0.76

¶ – Statistically significant at p < 0.05 level.

DC = Chiropractor

PCP = Primary Care Physician

## Discussion

In our study, clearly a majority of PCPs were willing to recommend that patients consult a chiropractor, yet they were reluctant to actually make a formal referral themselves. Only 30% of PCPs in our study and 50% in previous studies [16-18] have ever made a formal referral to a chiropractor. An important finding from our study is that when PCPs recommend chiropractic care, close to 88% preferred that patients contact a chiropractor on their own. There are several possible explanations for their unwillingness to "formalize" their relationships with chiropractors. PCPs fear malpractice litigations [19]. A perception that alternative care providers could be a threat to their practices could also have been an influence. Some of the PCPs in our study mentioned that they do not know enough about chiropractic to have an opinion, or do not view chiropractic as a legitimate health profession.

The lack of direct formalized referral relationships between PCPs and chiropractors has implications for efficiency, quality, and patient safety in the health care delivery system. It has been documented that allowing patients to contact other physicians on their own is likely to break continuity of care [20,21]. Patients are caught directly in the middle of this uncertainty between provider types. More research needs to be directed at better understanding of those issues surrounding the coordination and the loss of care which results from the poor professional relationships between these two provider types. This should include an examination of educational interventions to improve the documentation and sharing of clinical information and thereby enhance cross-disciplinary standards of care. Coulter et al in their study examined the inter-referral patterns between primary care physicians and alternate medicine providers and described a network system that can potentially provide an administrative structure to facilitate changes in physician attitudes in terms of referrals for their patients [7].

It is evident that the patients demonstrated a strong interest in chiropractic care. This finding is consistent with other studies [22,23]. Patient interest in alternate care is a great driving force for the PCP to refer or recommend them to chiropractors [22,23]. Several studies have revealed that general practitioners refer to alternate care providers for back pain, musculoskeletal conditions, and neck problems [21,23]. This is consistent with our finding that the most frequent reason for referral to a chiropractor was chronic musculoskeletal pain that does not respond to conventional treatments.

We found that PCPs in private practice are more likely to show positive referral attitudes towards chiropractors. It is likely that physicians in a private practice are not subject to the peer review or pressure in large institutional settings. Several authors have shown that physicians in solo practice are more open to unorthodox methods of treatment [23,24]. Also, Easthope et al [25] demonstrated that general practitioners in smaller practices view complementary therapies more favorably than those in larger practices.

A few studies have shown that age and sex influence physicians' perceptions of usefulness of alternate care therapies [26,27]. Sikand and Laken [26] have shown that younger physicians are more likely to exhibit positive attitudes towards complementary and alternative medicine. Glodsmitz et al[27] demonstrated that female general practitioners are more likely than their male counterparts to view complementary health care practices as useful. In contrast to these studies, sex and age were not significant predictors in our models, after adjusting for other model predictors. However, male physicians were more likely to have accepted referrals from chiropractors.

Our study adds precision to understanding the referral patterns of PCPs. A vast majority of PCPs seem to enjoy very good intra-professional relationships. However, it is clear that there is a major communication problem in inter-professional relationships with chiropractors. While chiropractic care has become popular with patients, PCPs in Iowa do not generally have a positive impression about chiropractic care and it is the case that MDs are very reluctant to make/receive referrals.

An issue to be considered is that our study assessed the views of primary care physicians in a predominantly rural state. It is quite possible that chiropractors play in different role in the primary care system of rural areas. Attitudes towards alternative care may differ between rural and urban physicians and between regions of the country. Hence, caution should be exercised in generalizing the study findings.

Finally, only 33% of the entire population of Iowa PCP physicians solicited to participate in this study responded to our survey and this raises questions about generalizability of our findings to all Iowa PCPs. While the information presented in Table 1 shows only minor differences between respondents and non-responders, there could still be some non-response bias attributable to unmeasured covariates. Previous studies requiring physician participation have encountered similar problems [28,29]. However, we should note that when compared to the general population, physicians constitute a relatively homogenous group. If our sample is representative, then the external validity of a study can still be achieved with relatively smaller sample sizes [29,30].

## Conclusion

PCPs enjoy very good professional relationships with other PCPs. However, the lack of direct formalized referral relationships between PCPs and chiropractors has implications for efficiency, continuity, quality, and patient safety in the health care delivery system. Further research must focus on identifying facilitators and barriers of developing positive relationships between PCPs and chiropractors.

## Competing interests

The author(s) declare that they have no competing interests.

## Funding

This research was made possible by funding by NIH-NCCAM – Project #AT-01-001 – Analysis of DC MDPCP Interprofessional Relationships. This investigation was conducted in a facility constructed with support from Research Facilities Improvement Grant Number C06 RR15433 from the National Center for Research Resources, National Institute of Health.

## Appendix 1

For the following questions, we define **formal referral**to mean that the referring provider facilitates the introduction or initial contact between the patient and a particular consultant.

1A

When you refer a patient to another medical doctor, do you more typically recommend that the patient contact the medical doctor on their own, or do you offer to initiate the contact with a **formal referral **from you to the other doctor?

1 I typically recommend that the patient contact the other medical doctor on their own

2 I typically offer to formally refer the patient to the other medical doctor

How many times have you **formally referred **a patient to another medical doctor during the past year? times

When you **formally refer **a patient to another medical doctor, how often does your referral include sending the following clinical information about the patient:

**Table 6:** 

	**Always**	**Usually**	**Sometimes**	**Never**
a) A case report	□	□	□	□
b) X-rays or X-ray report	□	□	□	□
c) Clinical records other than X-ray	□	□	□	□
d) Reason for referral	□	□	□	□

Other clinical information (please specify):

1B

Have you ever accepted a **formal referral ***from *another medical doctor? ★ Yes ★ No

If **"Yes"**, how many times have you accepted a formal referral from another medical doctor during the past year?

If "**Yes**", when you receive patients referred from another medical doctor, how often do you also **receive **the following clinical information about the patient?

**Table 7:** 

	**Always**	**Usually**	**Sometimes**	**Never**
e) A case report	□	□	□	□
f) X-rays or X-ray report	□	□	□	□
g) Clinical records other than X-ray	□	□	□	□
h) Reason for referral	□	□	□	□

Other clinical information (please specify):

If "**Yes**", when you receive patients referred from another medical doctor, how often do you **send **clinical information to the other medical doctor as a follow-up to their referral?

1 Always 2 Usually 3 Sometimes 4 Never

1C

Have you ever refused a **formal referral ***from *another medical doctor? ★ Yes ★ No

If **"Yes"**, for what reasons have you refused a referral from a medical doctor?

2A

Has a patient ever asked you for information about chiropractic? ★ Yes ★ No

Has a patient ever asked you to refer them to a chiropractor? ★ Yes ★ No

2B

Have you ever recommended to a patient that they might try seeing a chiropractor for their health complaint?

**(If NO, then check NO box and skip to next page) **★ Yes ★ No

If **"Yes**", how many times have you made such a recommendation during the past year?

For what type(s) of health complaints have you recommended that patients might try a chiropractor? (please list)

If **"Yes**", do you more typically recommend that the patient try a particular chiropractor or just any chiropractor in general?

1 I typically recommend a particular chiropractor

2 I typically recommend any chiropractor in general

If **"Yes **", do you more typically recommend that the patient contact the chiropractor on their own, or do you offer to initiate the contact with a formal referral from you to the chiropractor?

1 I typically recommend that the patient contact the chiropractor on their own

2 I typically offer to formally refer the patient to a chiropractor

For the following questions, we define **formal referral**to mean that the referring doctor facilitates the introduction or initial contact between the patient and a particular chiropractor

2C

Have you ever**formally referred **a patient to a chiropractor for evaluation or treatment? ★ Yes ★ No

(If No, then check NO box and skip to next page)

If **"Yes**", how many times have you **formally referred **a patient to a chiropractor during the past year?

If **"Yes**", for what type(s) of health complaints have you **formally referred **a patient to a chiropractor?

If **"Yes**", please indicate how often your **formal referral **to the chiropractor included the following clinical information about the patient.

**Table 8:** 

	**Always**	**Usually**	**Sometimes**	**Never**
i) A case report	□	□	□	□
j) X-rays or X-ray report	□	□	□	□
k) Clinical records other than X-ray	□	□	□	□
l) Reason for referral	□	□	□	□

Other clinical information (please specify):

3A

Have you ever accepted a **formal referral **from a chiropractor? Yes No

If **"Yes"**, how many times have you *received *a **formal referral **from a chiropractor during the past year?

If **"Yes"**, when you *receive *patients referred from a chiropractor, how often do you also ***receive ***clinical information about the patient?

1 Always 2 Usually 3 Sometimes 4 Never

If **"Yes"**, when you *receive *patients referred from a chiropractor, how often do you ***send ***clinical information to the chiropractor as a follow-up to their referral?

1 Always 2 Usually 3 Sometimes 4 Never

3B

Have you ever refused a **formal referral **from a chiropractor? ★ Yes ★ No

If **"Yes"**, for what reasons have you refused a referral from a chiropractor?

"Formal consultation" is a process whereby a healthcare provider refers a patient to another healthcare provider, e.g. for assistance with the diagnosis or treatment of the patient. The consultant reviews the patient's records, examines the patient, and formally documents his/her recommendations or plan for the patient's care.

"Curbside consultation" is an *informal *process whereby a provider (typically an MD) obtains information or advice from another provider (typically an MD) to assist in the management of a particular patient. The consultant neither reviews the patient's records nor examines the patient and does not document his/her recommendations.

3C

Has another **medical doctor **ever obtained clinical information or advice from you, by way of *informal *"curbside consultation"? ★ Yes ★ No

If **"Yes"**, how many times has this occurred during the past year?

Have you ever obtained clinical information or advice from another **medical doctor**, by way of *informal *"curbside consultation"? ★ Yes ★ No

If **"Yes"**, how many times has this occurred during the past year?

Has a **chiropractor **ever obtained clinical information or advice from you, by way of *informal *"curbside consultation"? ★ Yes ★ No

If **"Yes"**, how many times has this happened during the past year?

Have you ever obtained clinical information or advice from a **chiropractor**, by way of *informal *"curbside consultation"? ★ Yes ★ No

If **"Yes"**, how many times has this occurred during the past year?

3D

Do you consider your medical practice to be ***generalist***, ***specialist***, or ***both***. (circle one)

If specialist or both, please list your specialty here.

If both, what percentage of your practice hours are more oriented toward generalist practice. %.

What percentage of your practice hours consist of providing services that you consider "primary care" %.

## Pre-publication history

The pre-publication history for this paper can be accessed here:


